# Future Directions and Considerations for Talent Identification in Australian Football

**DOI:** 10.3389/fspor.2020.612067

**Published:** 2020-11-30

**Authors:** Nathan Bonney, Paul Larkin, Kevin Ball

**Affiliations:** Institute for Health and Sport, Victoria University, Melbourne, VIC, Australia

**Keywords:** Australian football, talent identification, ecological dynamic, skill assessment, small-sided games (SSG), kicking, AFL, talent selection

## Abstract

As the focus on the elite Australian Football League competition becomes greater so too does the demand for success. Clubs are heavily scrutinized for their draft selections and as such are taking more interest in the younger levels of competition in an attempt to identify and monitor talent. Based on contemporary talent identification knowledge, this review examines the current talent identification process in Australian football, with a focus on areas to potentially improve or inform future developments. Currently, a significant gap exists between static and isolated assessment procedures used to identify talent in Australian football and the dynamic nature of match play. Future assessments should consider factors such as maturation, fatigue and ecological dynamics. The addition of a valid and reliable technical skill assessment (e.g., a small-sided game) to the current Australian Football League draft combine was recommended.

## Introduction

Talent is a multi-dimensional concept and requires the effective and efficient organization of an individual's technical, tactical, physiological, and psychological competencies to be applied concurrently to meet the requirements of both the environment and the sporting situation (Vaeyens et al., 2009; Johnston et al., 2018). Talent identification programmes endeavor to discover this “talent” in individuals with the greatest potential to respond to a training intervention and reach the highest level in their sport (Hoare and Warr, 2000; Abbott and Collins, 2004). The ability to identify talented players in team sports is not only a financially rewarding business but a key component of future winning teams and long term success (Gee et al., 2010; Larkin and Reeves, 2018).

Talent pathways provide identified players with additional resources (e.g., expert coaching) and training to support the development of players to progress into high performance sport (Williams and Reilly, 2000). In Australian football, the talent pathway involves players progressing from regional development squads, to state squad selection, through to state and national academies with the eventual goal of being drafted into the Australian Football League (AFL). Talent pathways in Australian football have grown considerably with clubs now establishing their own youth academies. These academies aim to attract, retain and develop male and female players from 11 to 13 years (Tribolet et al., 2019) in an attempt to increase the talent pool, identify talent and select players onto their senior club list. Another way for players to showcase their talent is at the Australian Football League draft combine where physical, technical, and psychological capabilities are tested. These measures are then combined with talent recruiter subjective opinions to help identify playing status (i.e., elite or sub-elite) (Woods et al., 2015a) and select players (Robertson et al., 2015).

The specific traits assessed within talent identification programmes are varied. Key components of performance, such as the technical, tactical, physiological, and psychological, have all been used and examined within the literature (Johnston et al., 2018). These investigations; however, have delivered inconsistent results due to factors such as varying study designs, high variability (Johnston et al., 2018), unidimensional assessment designs (Bonney et al., 2019) or performance being constrained due to the task, environment and/or the individual (Newell, 1986). Based on contemporary talent identification knowledge, this review will examine the current talent identification process in Australian football from a physical (maturation; fatigue), technical, and tactical (i.e., decision making) perspective, while considering the associated theories (i.e., ecological dynamics; representative design) to potentially improve or inform future developments. It should be noted; however, there are many critical determinants of talent development including psychological, sociological, demographical, and socio-cultural influences (Woolcock and Burke, 2013; Toohey et al., 2018).

## Talent Identification in Australian Football

Australian football is an example of a team sport based upon many interconnected performance components (i.e., technical, tactical, physical, psychological). Each year, talent identified players are screened for their competency in each of these components. This screening occurs at a draft combine where players complete a testing battery of standardized technical (e.g., handball test), physical (e.g., 20-m sprint test), psychological (e.g., personality test), and medical tests (e.g., eye test) conducted over a 4-day period (Bonney et al., 2019). Results from these assessments are then used in combination with club recruiter opinion to justify talent selection decisions (Toohey et al., 2018).

Physical capabilities are commonly used in talent identification programmes (Johnston et al., 2018). When assessing drafted Australian football players, researchers discovered players who can perform above level-14 on the multistage fitness test and sprint 20-m in under 3 s were more likely to be drafted (Robertson et al., 2015). Furthermore, physical capabilities, such as aerobic capacity, speed and power have been used to distinguish between team selection and non-selection, career progression, and playing performance (Young and Pryor, 2007; Veale et al., 2010). Although physical testing does provide an insight into the physical abilities of potential draftees it does not identify how proficient a player is with the ball.

Match play performance requires a player to combine their technical, tactical, physical, and psychological components to effectively participate in the game. However, unidimensional assessment models are weak predictors of team selection and match performance. Gogos et al. (2020) investigated 1,488 drafted players between 1999 and 2016. They found physical and anthropometric testing results could only explain 4% of matches played and 3% of in-game performance measures with individual combine tests only explaining <2% of the matches played. The authors summarized these results by suggesting the Australian Football League draft combine is a poor measure of talent selection. This result is similar to research conducted in the National Football League (Kuzmits and Adams, 2008) and may suggest when tasks are completed in isolation, and low in representation of match play demands, their suitability to talent selection is limited (Bonney et al., 2019).

Whilst physiological capabilities are important in the game of Australian football, ball possession, and technical skill proficiency have been shown to be greater influences on match outcomes (Robertson et al., 2016). In addition, skill proficiency has been shown to have a higher level of reliability when attempting to identify talent (Gabbett et al., 2007a). For example, in a study of junior volleyball players it was found particular skill test results of game proficiency (subjective coach evaluation of passing and serving) were the only variables to discriminate between selected and non-selected players in comparison to physiological or anthropometric results (Gabbett et al., 2007a). A similar result was found in rugby players. When investigating first grade rugby players Gabbett et al. (2007b) found selection was based more upon playing experience and skill proficiency of players than their physical capacities. The authors did; however, note a high level of physical fitness did contribute to effective playing ability in these selected players. In Australian football, the analysis of skill effectiveness has been successful in identifying first round draft picks in comparison to second and fourth round picks. First round draft picks had more kicks, effective disposals, contested possessions, and contested marks than athletes selected later in the draft selection (Woods et al., 2017). Furthermore, drafted players were able to deliver the ball more times within the attacking 50-meter (50 m) zone than non-drafted players (Woods et al., 2016a).

Sport specific skill proficiency has also been shown to be a sensitive predictor of successful teams (Kempton et al., 2017). When comparing winning to losing a game, it was discovered winning quarters consisted of more skill involvements (i.e., kicking and handballing) and higher skill efficiency whilst quarters lost involved more physical requirements (Sullivan et al., 2014a). This highlights the importance of skill involvements and skill proficiency, in comparison to physical activity profiles, to team success. Although the current technical assessments used in the Australian Football League draft combine can potentially predict talent it must be considered that the assessments used (i.e., the Australian Football League kicking efficiency test) are predominantly performed in an isolated setting, separating the skill from the demands of match performance. Consequently, these assessments may lack the identification of key components such as decision making, game tempo adjustment, and tactical awareness (Burgess and Naughton, 2010). Due to the scarcity of representative validity within these assessments it is plausible to suggest the results obtained may not accurately identify all talented players and may produce results different from match play. As such, talent identification models need to provide opportunities for athletes to develop these underlining skills and have these skills monitored throughout their involvement in the programme as well as adjusting training loads accordingly (Abbott and Collins, 2004; Abbott et al., 2005; Burgess and Naughton, 2010).

When contemplating talent identification in Australian football, it is apparent more sport specific research is required to obtain clarity on the interconnecting components. Researchers have used multidimensional assessment designs (e.g., isolated assessments of technical, physical, and psychological capabilities) to identify talent (Woods et al., 2016c; Tribolet et al., 2018). Although these designs were more successful than single assessments, they do not consider how these components are interconnected or replicate the performance demands of the game. More recently, Bonney et al. (2020d) developed a small-sided game where all the performance components were combined into the one assessment. This assessment was 97% successful in identifying talented youth players and was suggested to be a more time efficient and ecologically valid way to identify talented players. Reviews of talent assessment; however, have highlighted the high level of variability in the elements separating higher and lesser skilled players (Johnston et al., 2018). Although, variability should be carefully considered as variability can be “good” (functional) and “bad” (dysfunctional) (Woods et al., 2020b). A possible suggestion to achieving greater continuity is to have studies based on sound theoretical principles and valid research designs (Bergkamp et al., 2018).

Current research suggests sports training and assessment environments should be guided by an ecological dynamics framework (Davids et al., 2013b; Bonney et al., 2019; Woods et al., 2020a,b). This framework may be used to guide the design of new assessments by practitioners developing assessments centered around the athlete-environment interaction (Woods et al., 2020a). These assessments should consider the interacting constraints, movement behaviors, contain adequate environmental variables and ensure the functional coupling between perception and action processes (Pinder et al., 2011). Additionally, they should consider the effect of physical and psychological maturity and relative age effect (Burgess and Naughton, 2010) whilst challenging players to make accurate and timely decisions whilst executing the skill under some level of fatigue (Dawson et al., 2004; Gonzailez-Va-llora et al., 2015).

The ability to proficiently kick the ball under match performance demands (e.g., evading opponents and kicking the ball to a teammate) is a critical factor in Australian football. A coach's perception of player performance is largely influenced by the number of disposals they have had and their effectiveness to pass the ball and maintain possession (Sullivan et al., 2014b). Considering the importance of kicking in Australian football, it is surprising there is currently very little research conducted on the application of using kicking performance as an assessment tool for talent identification (Cripps et al., 2015; Woods et al., 2015b). In an attempt to assess kicking skill performance of elite youth Australian football players, the Australian Football League included two skill tests to the Australian Football League draft combine; however, the ecological validity of both tests is a major concern (Bonney et al., 2019). The most representative skill test at the Australian Football League Combine is the set-shot goal kicking test. A set-shot is a closed skill performed during a stop in play as a result of a mark or free-kick penalty. To the author's knowledge no research has been conducted on this test to validate its use and without established validity it is unclear whether the test measures what it claims to Larkin et al. (2014). The second assessment, the kicking efficiency test, involves a player running toward a feeder who receives the ball and delivers it to one of six randomly assigned stationary targets (Cripps et al., 2015). Kicking performance is subjectively assessed on a scale from 0 to 5 (5 being the highest score) for each kick. Cripps et al. (2015) investigated the test on 121 semi elite under 16 (U16) male players and found the inter-rater reliability to be high; however, the test could only differentiate between dominant and non-dominant kicking leg accuracy across varying distances. The authors concluded more research was required to determine if the test can distinguish between higher and lesser skilled players and if kicking ability changes with age (Cripps et al., 2015). Woods et al. (2015b) also assessed the kicking efficiency test on 50 U18 male players (25 state representatives and 25 non state representatives) and found when kicking accuracy and ball speed were combined playing status was able to be predicted. A limitation of the current kicking test is the assessment is conducted in isolation and does not assess the range of in-game kicking constraints typically performed within the performance environment. As such, the dynamic interactions between organismic, environmental, and task constraints are not necessarily representative of the requirements of match play leading to possible invalid results (Newell, 1986; Abbott et al., 2005; Pinder et al., 2011; Vilar et al., 2012).

Researchers have attempted to provide greater clarity on skill assessments through the suggestion of a 5-Level Performance Assessment Model (Bonney et al., 2019). The model applies match play notational analysis to separate technical game skill on a continuum comprising of Level-1 (i.e., laboratory test), Level-2 (i.e., static field-based test), Level-3 (i.e., dynamic field-based test), Level-4 (i.e., small-sided game field-based test), and Level-5 (i.e., match play). The proposed model provides coaches with a better understanding of the potential performance demands and key outcomes associated with each level. In an attempt to fill the void between Level-2 and Level-5 researchers have developed the first two valid and reliable Australian football kicking skill assessments: a dynamic kicking assessment (an example of a Level-3 test) (Bonney et al., 2020c) and a small-sided game (an example of a Level-4 test) (Bonney et al., 2020d). These assessments used notational analysis to consider match play kicking demands and applied a more integrated approach of match play components. Interestingly, the authors found both the dynamic kicking assessment (68.3% successful) and the small-sided game (97%) were successful at identifying player skill levels. The results may suggest as assessments move further along the continuum, integrating more components of match play, they are also more successful at identifying talent. It was proposed these results may provide coaches with worthwhile information regarding player kicking performance during competition rather than specific details on how they will perform the skill. As such, Level-3 and Level-4 assessments (such as the ones discussed above) may provide greater insights into match play kicking proficiency thereby improving current talent identification programmes. Accordingly, these assessments are recommended to be included in future Australian football draft combines.

## Maturation

Maturation is an important factor to be considered when trying to identify talent. When identifying athletes below the age of 15, differences as small as 1-year in stages of puberty can have a significant effect upon an athlete (Cobley et al., 2009). Early maturers have more developed physical attributes such as height and weight which are related to a player's strength, power, and speed (Russell et al., 1998; Sheppard et al., 2012). As such, these players appear more competent than their peers and consequently more likely to attract the attention of talent recruiters (Cobley et al., 2009; Figueiredo et al., 2010). Coutts et al. (2014) investigated 806 players drafted to play elite Australian football. They found drafted players were more likely to be born in the first quartile and first half of the year, had advanced physical and psychological maturity and had exposure to higher level coaching.

The term relative age effect (RAE) is used to describe a selection bias dependent upon the time of year the athlete is born (Andronikos et al., 2016). The relative age effect appears to be most pronounced between the ages of 15 and 18 at representative level (Cobley et al., 2009). Haycraft et al. (2018) examined the influence of relative age effect across the Australian Football League talent pathway. They found a relative age effect was present at the state U16 level and is maintained at State and National level combines. Collectively these studies would imply early maturers are being selected based more upon physical statue rather than skill proficiency.

In soccer, stabilization in physical activities, such as repeated sprint ability, do not occur until 18-years of age (Spencer et al., 2011). As such, trying to identify players on physical abilities prior to this age was unpredictable. Interestingly, once all athletes have gone through maturation, it has been found the late maturers are more likely to reach the elite level (Pearson et al., 2006), achieve longer careers and potentially have greater financial contracts (Gibbs et al., 2012; Deaner et al., 2013). This phenomenon has been identified as the “underdog” hypothesis, whereby younger players overcome early disadvantages (e.g., non-selection in early stages of a player's career) to become elite players (Schorer et al., 2009; Gibbs et al., 2012). This is largely due to once they have caught up to the early maturers physical statue, their years of playing against bigger and stronger competition have enabled them to develop superior technical proficiencies, tactical awareness (Schorer et al., 2009; Gibbs et al., 2012) and psychological advantages (Cumming et al., 2018).

It is important selection does not discriminate against late-maturing players who may develop their abilities later. A suggestion to avoid this is for coaches to apply a more skilled based selection criterion (e.g., skill proficiency) and look toward long term athlete development rather than short term success (Cobley et al., 2009). Cripps et al. (2016) investigated the effect of maturation on technical skills on 94 under 16 Australian football players. They found no significant differences between early and late maturation groups when examining technical skills; however, coaches' subjective opinion of overall technical skill, marking, and ball winning abilities were advanced in the earlier maturing group. In a follow-up study, Cripps et al. (2019) investigated if development pathway coaches' were influenced by the maturational status of adolescent players when predicting long-term career attainment predictions. The authors found predictive accuracy to be greatest for the late maturing player (79%) in comparison to early maturing players (52%), highlighting the complexity associated with identifying players at early stages of development pathways. These results have been supported from investigations in other sports. Figueiredo et al. (2010) noted how soccer-specific skills performed by players 11–14-years old were less affected by maturation than physiological attributes. Whilst in handball (Matthys et al., 2012) and basketball (Silva et al., 2010) sport-specific skill appeared to be independent of pubertal status. Overall, these results suggest technical skill assessments may be less influenced by maturation and subsequently a more appropriate way to identify and select talent in youth Australian football.

## Fatigue and Pressure Situations

Successful athletes not only display a high proficiency of technical skill and decision making ability but they can display these traits under both fatigue and pressure situations (Kitsantas and Zimmerman, 2002; Royal et al., 2006). Fatigue; however, is an element often neglected by skill tests. There are many opinions on how to explain fatigue such as a decline in muscle force (Komi and Tesch, 1979), an increase in metabolic accumulation (Hargreaves et al., 1998) or energy substrate depletion (Balsom et al., 1999). These opinions can be further examined according to the time period where the activity took place. For example, fatigue following an intense period of exercise appears to be related to disturbances in muscle ion homeostasis whilst the initial constraint in generating maximal force post half time may be due to a lower muscle temperature; with the fatigue experienced toward the end of the game due to low levels of glycogen (Mohr et al., 2005).

Due to the varying definitions of the word “fatigue” researchers have suggested each definition has its own place in the literature dependent upon the context in which the term is being used (Abbiss and Laursen, 2007). During the game of Australian football, fatigue can lead to many detriments in playing performance. Kicking performance may decline due to alterations in the neuromuscular systems and force generation capacity, which may alter the mechanics of the kicking performance (Kellis et al., 2006). More recently, it has been shown elite level players adjust their movement strategy when kicking “fresh” compared to when they are fatigued (Coventry et al., 2015). Furthermore, fatigue can impact the speed at which information is taken in, processed and a response is initiated thereby impeding a player's ability to effectively select the correct passing option and execute the skill proficiently. Finally, moderately to high fatigue can impair performances requiring strength, endurance, and rapid movements (Lidor et al., 2009) such as those displayed in Australian football marking, tackling, running and kicking.

When comparing experienced and less experienced players in Australian football, the experienced players had more skill involvements during the second and fourth quarters (in peak 3-min block) and performed technically and physically better following peak periods of match play than their counterparts (Black et al., 2016). A possible reason for this may be the shorter break duration at quarter and three quarter time, with lesser skilled players having more residual fatigue in their bodies following these breaks (Abbiss and Laursen, 2007). This research highlights how important skill proficiency is when not only in a fresh state but also, if not more importantly, in a fatigued state.

The type of activities players participate in will have a large impact upon their performance. Activities such as tackling and collisions have been shown to significantly impact the number of repeated high intensity efforts performed (Gabbett, 2013). In a study of 24 elite Australian football players it was shown less experienced players were more likely to cover less high-speed distances during the period following the most intense passage of play in comparison to experienced players (Black et al., 2016). Furthermore, these less experienced players had fewer involvements during high-intensity periods of the match as well as during the periods subsequent to the most intense periods of play. Joseph et al. (2017) investigated the relationship between aerobic capacity and kicking performance in Australian football. They noted players who had a higher yo-yo intermittent recovery two score had higher kicking speeds and better accuracy scores than players with lower aerobic capacities. This research is important as it demonstrates as fatigue increases technical and tactical abilities may deteriorate and this regression is more prominent in less experienced players than experienced players.

Many sport skills are performed in a fatigued state. Research; however, has suggested match related fatigue has a greater effect on a players ability to get involved with the ball than it does on a player's skill proficiency (Rampinini et al., 2009). The intensity of the fatigue is an important factor to consider within this context. In a study of 20 college students it was found soccer passing performance was better following moderate muscle fatigue in comparison to rest or high levels of fatigue. This test; however, was completed in a static environment where players had to dribble the ball within a certain area and pass the ball to a stationary box with no involvement from “live” opponents (Lyons et al., 2006). The level of the athlete may also be an important consideration. In water polo it was shown elite athletes are more resilient to physiological stresses occurring during competition and as such are better able to maintain their technical accuracy. These elite water polo players made 18% better decisions with high fatigue than with low fatigue with shooting accuracy and velocity being unaffected (Royal et al., 2006). Shooting technique; however, did decrease by 43% between pre-test and high-fatigue suggesting elite athletes are able to self-regulate in order to optimize their performance (i.e., they decreased their proficiency on non-essential technique to ensure accuracy and speed was maintained) (Kitsantas and Zimmerman, 2002; Royal et al., 2006). As current Australian football talent identification skill tests do not cater for fatigue, it is suggested a new skill test is implemented, where the level of fatigue is appropriate for the age and ability level of the athlete. Players can then be assessed on their ability to obtain possession of the ball, make decisions and execute a skill under fatigue conditions. The information gained from such a test may provide critical details to coaches regarding a player's technical skill performance during competition, rather than specific details on how a player will execute the skill.

## Decision Making

When athletes move, they do so with purpose. They carefully consider the information available in their environment, establishing their options and likely outcomes, before progressing (Araujo et al., 2006). Elite team sport players are therefore expert decision makers with the ability to read the play and make timely and accurate decisions (Berry et al., 2008). Furthermore, these attributes can take up to 13 years/4,000 h of invasion-type activity and sport specific practice to obtain (Baker and Cote, 2003; Berry et al., 2008). In knowing this, sport practitioners have used decision making assessments to identify talent.

In an attempt to assess decision making ability in Australian football players, video-based decision making tasks have been examined (Lorains et al., 2013; Woods et al., 2016b). When investigating 85 male Australian football players, across three skill levels (elite, sub-elite, and novice), it was found elite players have superior decision making accuracy compared to sub-elite and novice players (Lorains et al., 2013). Furthermore, it was suggested elite players use superior processing efficiency to perform more accurately when the speed of the video is increased from 0.75 speed to 2.0 speed. Similar results were also found when investigating the decision making ability of U18 Australian football players. Results indicated a video-based decision making task could correctly classify 92% of talent-identified players and 72% of the non-identified players, with the talent-identified players making more accurate game-based decisions compared to non-talent-identified players (Woods et al., 2016b). More recently, 360 degree virtual reality footage has been used as an alternative off-field decision making assessment tool to match broadcast; however, more research is needed to clarify its application to Australian football players (Kittel et al., 2019, 2020).

When trying to decipher how a player makes a decision it is crucial to examine the environmental and task constraints present, as these two components have an integral effect on the decision making process. A model used by practitioners to examine decision making ability is the constraints-led approach. This approach is an ecological model, centered on the relationships that emerge from interactions of players and their performance environment (Renshaw et al., 2016). It requires the practitioner to identify and modify interacting constraints to facilitate the emergence of perception-action couplings (Renshaw et al., 2016). To replicate match play decision making, it is critical these same constraints are present otherwise movement patterns may alter and the decision making process will adjust in order to adapt to the different stimulus (Araujo et al., 2006). To enhance decision making ability players use specific stimuli from the environment to inform their movement decision. In cricket, less skilled players spent more time fixating on distal cues, such as the ball hand area, whilst skilled players fixated on more proximal predictive cues such as the bowling arm, trunk-hips and predicted ball-release area (McRobert et al., 2011). Interestingly, both groups improved when more contextual information was available. In addition, players may also use cues such as postural movements of opponents and task specific structures or patterns to make accurate decisions (Roca and Williams, 2016). This research suggests how practice needs to be representative of the actual task and the context constraints in order to elicit enhanced player decision making (McRobert et al., 2011).

Search strategy has been suggested to differentiate between skilled and lesser skilled players. Roca et al. (2011) found skilled soccer players were able to search the environment more effectively and efficiently than lesser skilled players. Although they search the entire situation they are more focused on informative locations assisting them in making more accurate decisions in a timelier manner (Roca et al., 2011). The ability to successfully achieve this in a quick and unconscious manner is trainable; however, it takes time to build (Roca et al., 2011). An interesting observation by Berry and Abernethy (2009) outlined how elite Australian football players felt much of the training environment is not effective enough at developing perceptual and decision-making skill to a level where it meets the demands of match play. This provides an important consideration when assessing talent. When determining a player's ability to play at the highest level, technical skill assessments need to ensure they are challenging a player's ability to perceive information from the environment, process this information, make a decision and execute a skill under the same demands as match play. It is therefore critical recruiters and coaches understand the identification of talented athletes will be limited in value unless information regarding the proficiency of skills of the game are considered with the ability to read the game and anticipate an opponent's intention (Reilly et al., 2000). This is supported by Cupples and O'Connor (2011) who investigated performance indicators in elite youth rugby from a coach's perspective. They discovered cognitive ability to be the greatest influence of playing position in elite youth rugby league players, followed closely by game skills and finally to a lesser extent physiological indicators. It was further discussed how the development of a skills test, simulating game specific conditions, will improve the coach's knowledge of player strengths and weaknesses, whilst providing performance criteria for the monitoring of improvements throughout the season or longer (Gabbett, 2002; Cupples and O'Connor, 2011). Accordingly, the current skill assessments utilized within the Australian Football League draft combine should be reviewed with more match specific skill assessments implemented (e.g., requiring players to make decisions and execute skills under representative conditions).

## Ecological Dynamics

Ecological dynamics is a framework researchers use to understand and explain sport performance (Seifert et al., 2017). This framework is based on human behavior and motor learning understandings to underpin the learning design in the performance context (Davids et al., 2012; Seifert et al., 2017). There are three main components of ecological dynamics. The first is viewing players and teams as a complex adaptive system, the second involves cognition and behavior being considered together and the third component relates to how behaviors are organized based upon the information available (Seifert et al., 2017). In comparison to other methodologies (e.g., perceptual-cognitive training), ecological dynamics requires all parts of the system (brain, body, and environment) to be dynamically integrated (Renshaw et al., 2018). Alternative methodologies assume perceptual and cognitive systems and brain processes can be trained in isolation from the informational constraints of competitive performance environments (Renshaw et al., 2018). Although training these systems and processes in isolation can be an efficient use of time and money, current research suggests there is little evidence to support their effectiveness (Harris et al., 2018). Accordingly, sport scientists have suggested the use of this framework to improve the analysis of data collected and to gain greater insights into competitive performance behaviors (Travassos et al., 2013; Browne et al., 2019).

Ecological dynamics is an important consideration in the assessment of performance as player skill acquisition and tactical behaviors are constrained by player task constraints (Silva et al., 2014). During competition players are constantly changing their behaviors based upon the constraints imposed by other players and the environment (Araujo et al., 2006). For example, when executing a kick in Australian football, players need to consider the movement and speed of their teammates, the movement and speed of the opposition, who they will kick the ball to, the type of kick to be executed, the amount of force to be applied to the ball and the amount of time they have to execute the kick. Woods et al. (2020a) provided a practical example of how to apply ecological dynamics to the performance preparation of Australian football players. This framework was called “Heads Up Footy” and considered the individual environment, self-regulating and adaptable foundations of ecological dynamics. Although advancements have been made in developing the relationship between a player and their training environment (Woods et al., 2020b), further considerations are required when selecting and designing performance assessments. The ecological dynamics framework is therefore an important factor to consider when developing performance assessments to ensure players are afforded enough information to achieve the goal of the task (Araujo et al., 2006).

When designing performance assessments, the opportunity players are afforded to perform actions should be based from a specific performance context (Araujo et al., 2006; Woods et al., 2019). Factors to be considered in the performance context should include the interactions between players and teams, notational analysis of game performance and time motion (Travassos et al., 2013; Bonney et al., 2019). When attempting to assess player performance the assessment should ensure the same intentions, information and behaviors are available and performed (Davids et al., 2012). Additionally, these assessments should create a context where the decision making behavior is largely anticipatory based on the availability of information from the player's actions and their environment (Araujo et al., 2006). These performance contexts can then be used to assess a player's ability to recognize affordances for action and execute their skill (Davids et al., 2012).

Current Australian football methods of technical match play assessment are largely focused on recording frequency of actions and patterns of player's movements (Travassos et al., 2013). These approaches are too centered around discrete actions, considered in isolation from the game context and do not consider the interpersonal interactions between players and teams (Travassos et al., 2013). Match play constraint interactions are an important consideration as they can influence technical skill proficiency. For example, an Australian football players kicking proficiency average may be 54%; however, when the player kicks the ball in under 2 s and over 40 m the proficiency may decline to 47% (Browne et al., 2019). As such, when constraints are viewed from a more integrated manner greater insights into player behavior performance can be found (e.g., kicking performance of players). The use of the ecological dynamics framework, to understand these interacting constraints, may also assist in the identification of key events which can be replicated in training and performance assessments (Couceiro et al., 2016). Therefore, to achieve a greater understanding of how a player may perform under match like conditions a more representative performance assessment, where more constraints can be applied to the one task (e.g., time, distance, and pressure), may be more appropriate for talent identification in Australian football.

## Representative Design

The main aim of a performance evaluation assessment is to demonstrate how the assessment relates to the competitive environment (Davids et al., 2013a). To increase the likelihood of the assessment session to be representative of the competitive environment, researchers have suggested the use of a “representative learning design” as a theoretical framework. This framework suggests testing and practice sessions should be reflective of match play conditions including the technical and tactical execution of skills (Corbett et al., 2018) and the environmental conditions, actions, and perceptual stimuli present during the competitive environment (Davids et al., 2005, 2013a; Araujo et al., 2006, 2007; Pinder et al., 2011; Vilar et al., 2012).

Team sport athletes are well-rehearsed with intended movement patterns. These movements; however, cannot be entirely planned and acted upon due to the unpredictable environmental elements and constraints (i.e., opposition movement) (Chow et al., 2011). Therefore, movements and decisions are largely anticipatory in nature based upon key information from their actions and the external environment (Araujo et al., 2006). When assessing the anticipatory visual cues for 25 tennis players (13 skilled and 12 novice) it was found skilled players were more accurate with live and video displays (but not with point-light displays) than novices (Shim et al., 2005). This research highlights the importance of visual perception (i.e., opposition movement) in the assessment task and how this can effect skill execution.

A player's ability to effectively move is dependent upon the environment, perceptual stimulus, goal of the task and/or constraints present (Pinder et al., 2009). If alternative movement patterns are implemented, dissimilar outcomes may occur leading to an ineffective assessment of the skill. Static assessments lack functionality and do not successfully represent the constraints of the performance environment (Pinder et al., 2009). In a study analyzing the practice and free-throw performance of male Division 1 basketballers, it was shown practice free-throw percentage (74.5%) was higher than game percentage (69.2%) (Kozar et al., 1995). Free-throw shooting is typically practiced in a block modality, whereas in a game a free-throw is typically taken twice in succession. However, when only the first two practice free-throws were accounted for the percentage decreased to 69.8% which is comparable to match play performance (Kozar et al., 1995). In another study of seven team coaches in the Federation of International Hockey 2011 Champions Trophy tournament (field hockey) it was identified six of the coaches used more match representative designs when constructing their field goal shooting practice (Slade, 2015). The coaches found by moving away from the closed skill drilling sessions, into a more match representative task design, players were able to improve their tactical understanding, decision making and likely player patterns when attempting field goal shooting (Slade, 2015).

The development and implementation of a skill assessment should be carefully considered. Renshaw et al. (2007) investigated the differences in cricket batting actions when facing a human bowler in comparison to a bowling machine. Four 21-year old premier league cricket participants demonstrated how changing from a human bowler to a bowling machine instigated a re-organization of the coordination and timing of the forward defensive shot (Renshaw et al., 2007). Similar results were also found in a follow up study were a change in movement pattern occurred when batters faced a real bowler compared to a bowling machine (Pinder et al., 2009). The findings showed when facing a bowling machine participants modified their peak height of backswing, drive initiation of the downward swing, front foot initiation, and front foot placement (Pinder et al., 2009). Careful consideration therefore needs to be applied to designing assessments for athletes to complete. Assessments should be dynamic and consider the perceptual information being presented to ensure the movement patterns performed are representative of those experienced during match play (Pinder et al., 2011). It could be argued these more random situations more closely resemble the dynamic nature of sport allowing the athlete to practice under match like conditions thereby facilitating a more effective transfer of skill into match play (with the same concept being applicable to skill assessment sessions) (Farrow, 2010).

Australian football is played in an open environment. Within this setting expert players are constantly adapting to their changing environment to perform consistently (Araujo and Davids, 2011); however, current Australian football skill assessments are conducted in a static environment. These environments are not effective in assessing skill proficiency as they simulate behaviors different to that performed in match play (Pinder et al., 2011). Furthermore, these settings eliminate the ability of the athlete to demonstrate their competence in anticipation, problem solving and attention, which consequently affects their ability to execute their skill proficiently (Falk et al., 2004; Lidor et al., 2009). Skill assessments should create opportunities for players to trial moving in a variety of different patterns. This would demonstrate the player's ability to overcome constraints during match play in order to achieve the intended performance outcome (Davids et al., 2013a). Active opponents are integral to this concept as they assist in the environment being more dynamic and challenging for the players to perform within (Vilar et al., 2012). A significant gap therefore exists between current static assessment procedures used to identify talent in Australian football and the dynamic nature of match play. A new representative skill assessment in the Australian football draft combine, where behavior emulates competition (e.g., small-sided games), may assist in abridging this void.

## Small-Sided Games

Match play is unstable, dynamic, and unpredictable. There is a strong need for coaches to develop assessments and drills where the components of match play are more interconnected whilst replicating the most intense performance demands of competition without a decrease in running performance (Johnston et al., 2015). Well-designed open skill assessments not only provide a suitable amount of skill affordances but are more contextually relevant to the game setting (Farrow et al., 2008). When examining the skill and physiological demands of open and closed training drills, in elite junior Australian football players, it was highlighted how open skills were more physically and cognitively demanding than closed drills (Farrow et al., 2008). Australian football requires a high level of physical ability in combination with technical skill and decision making ability. The application of open skill assessments (e.g., small-sided games) are appropriate as they provide a suitable amount of skill affordances and are more contextually relevant to the game setting than traditional assessment methods (Farrow et al., 2008).

Small-sided games demand a greater cognitive effort (Lee et al., 1994) which is associated with greater decision making capabilities, skill execution (Turner and Martinek, 1999), and tactical awareness (Chatzopoulos et al., 2006). In addition, small-sided games challenge the athlete to make timely decisions whilst proficiently disposing of the ball in a simulated match environment (Davids et al., 2013a; Young and Rogers, 2014). Gabbett (2009) investigated the application of using small-sided games for improving skill and physical fitness in team sport athletes and found they were effective in developing technical and perceptual expertise. In addition, small-sided games have the ability to challenge a player's agility, speed, aerobic conditioning and power (Bujalance-Moreno et al., 2018). Small-sided games can be manipulated in a variety of ways to achieve the desired outcome. For example, coaches will vary environmental conditions such as the playing area, practice objectives, and rules of play to ensure players are performing enough skill executions (Davids et al., 2013a). Bonney et al. (2020a) investigated the influence of manipulating player numbers (i.e., 5v5, 5v6, 6v6, 7v7) in an Australian football small-sided game. They found different player numbers produced different technical and physical responses. For example, the 5v6 condition produced more technical events under more pressure situations, balanced numbers produced more contested possessions and lower player density conditions produced higher physiological responses.

Assessments of playing performance can be accurately assessed within match play situations. When a representative environment allows an athlete to display their tactical understanding and their ability to make timely and accurate decisions, combined with their ability to proficiently execute game related skills, players can be accurately identified as either higher skilled or lesser skilled (Piggott et al., 2019; Bonney et al., 2020d). Researchers have also investigated if small-sided games can discriminate perceptual-cognitive-motor capability and predict disposal efficiency in match performance of skilled Australian football players (Piggott et al., 2019). The participants played three small-sided games for 3 min with each disposal from only the attacking players scored for decision making and motor skill execution. The authors found the higher skilled players were able to make better decisions and obtain a higher scores than the lesser skilled players. Furthermore, only the higher skilled players scores were successful in predicting disposal proficiency in match performance (Piggott et al., 2019).

The use of constraints (e.g., two consecutive touches per possession) in small-sided games can enable the task to more accurately replicate match play (Almeida et al., 2012). Furthermore, the constraints applied can encourage players to explore solutions to problems (Cronin et al., 2017) highlighting player's strengths and areas of improvement. It is important for players to develop their ability to impact match play as player and team impact scores have been shown to not only have a significant correlation with winning but also as a valid method of assessing game performance (Heasman et al., 2008). Interestingly, the main indicator correlating with the derived team impact score was effective long kicks (Heasman et al., 2008) with previous research finding similar results (Stewart et al., 2007). It is important therefore, this type of kick is accurately assessed within any future Australian football talent identification battery assessment.

The size of the small-sided game perimeter is varied in the literature. In Australian football playing areas such as 30 × 20 m, 45 × 30 m, 23.2 × 20 m, 30 × 40 m, and 40 × 50 m have all been used to compare the physical and technical demands of small-sided games in elite Australian football (Davies et al., 2013; Fleay et al., 2018). Davies et al. (2013) demonstrated how a reduction in players resulted in increased amounts of variability seen during training combined with small increases in total agility maneuvers. In another Australian football study, Fleay et al. (2018) found “large” small-sided games generated fewer technical errors and tackles, more bounces and a greater physical activity profile in comparison to “small” and “medium” dimensions. An earlier futsal study found similar results (Duarte et al., 2009). The authors noted when there was a decrease in the number of players from 4v4 to 2v2, with a pitch size of 20 × 20 m, player intensity increased and more frequent tactical actions occurred. It was hypothesized this was due to more surface area being available per player (Duarte et al., 2009). In contrast to these two studies, a rugby league study analyzing the effect of field size on the physiological and skill demands of players involved in small-sided games noted no significant skill involvement differences when using a 10 × 40 m playing area vs. a 40 × 70 m playing areas (Gabbett et al., 2012). Increases in meters traveled and distances covered at moderate, high and very high intensities were; however, noted in games played on the larger field size, with senior elite players recording higher amounts than junior elite players. The authors suggest the difference in small-sided game field size in rugby league may have a greater impact on physical output than volume or quality of skill executions (Gabbett et al., 2012). These results suggest when implementing a small-sided game, as a method of talent identification, careful consideration should be given to the area afforded to players to ensure the technical and physical outputs produced are appropriate to the task goals.

Small-sided games are commonly used as a method of assessing and preparing players for match play. Coaches; however, should be mindful of how the constraints manipulated in these games compare to match play. When comparing an Australian football 5v6 small-sided game to Australian football match play, small-sided games have been shown to produce higher kicking proficiency, number of kicks executed, meters traveled per minute and percentage of high intensity running (Bonney et al., 2020b). In comparison, players had less time affordance to execute a kick and achieve higher maximum running velocities during match play (Bonney et al., 2020b). In soccer, researchers have found match play to produce greater player total distance covered per minute, total number of duels and lost ball possessions (Dellal et al., 2012). Furthermore, fewer ball touches (i.e., 1 or 2) increased the difficulty for players to perform technical actions. The authors continued to suggest if one or more of the physical, tactical, technical components can be developed simultaneously rather than in isolation, coaches would have the opportunity to maximize their contact time with players and increase efficiency of their training sessions (Dellal et al., 2012).

Overall, these studies indicate by changing the pitch area, the amount of players participating and constraints by which the players are abiding by, the intensity of the game can be modified. For example, a smaller number of players (lower player density) can produce higher external and internal loads (Randers et al., 2018). Furthermore, when fewer player numbers are used with a large pitch size players work at a higher exercise intensity (Hill-Haas et al., 2011). Considering small-sided games are the closest representation of match play conditions from a physiological, tactical, and technical perspective and player performance should be analyzed from within a simulated, competitive environment it appears small-sided game testing is the best solution (other than actual match play) for assessing competition skill performance (Bonney et al., 2019).

## Conclusion

As the focus on the elite Australian Football League competition becomes greater so too does the demand for success. Clubs are scrutinized for their draft selections and as such are taking more interest in the younger levels of competition in an attempt to identify and monitor talent. This article discussed the current assessments used in the Australian football talent identification pathway and provided future directions and considerations to more accurately and efficiently identify talent. Currently, a significant gap exists between static and isolated assessment procedures used to identify talent in Australian football and the dynamic nature of match play. Future assessments need to consider the effect of maturation and fatigue on the performance outcome. The addition of a valid and reliable technical skill assessment (e.g., small-sided game) to the current Australian Football League draft combine was recommended. Such assessments may provide greater insights into match play kicking proficiency as players can be assessed on their ability to obtain possession of the ball, make decisions and execute a skill. Results from player performance can then provide critical information to coaches regarding a player's technical skill performance during match play, rather than specific details on how a player will execute the skill.

## Author Contributions

NB: conceptual idea and writing of article. PL and KB: conceptual idea and reviewer. All authors contributed to the article and approved the submitted version.

## Conflict of Interest

The authors declare that the research was conducted in the absence of any commercial or financial relationships that could be construed as a potential conflict of interest.
